# The Nurse Who Thinks

**Published:** 1919-10-04

**Authors:** 


### THE MATRONS' AND SISTERS' DEPARTMENT.

### THE NURSE WHO THINKS.

Which of us has not been pulled up sharp many times in 'prentice days with the remark, "It's no business of yours to think?" Unquestioning obedience to orders, absolute concentration on the method rather than on the aim of the process, these are the qualities esteemed in the learner, and it is not to be denied that the effort of ratiocination is too apt to interfere with the required precision. Perhaps it is not in the act of taking aim at a partridge that the sportsman's mind should be filled with theories about parabolic curves, but there is need in the gunner's course for science. There can hardly be two opinions about the fact that the training of probationers, as carried on in many institutions, actually discourages independent thought or reasoning about the duties and arts which have to be mastered. We might go a step farther and assert that this discouragement of the probationer's mental powers has resulted in the manufacture of a vast number of unthinking, thoughtless nurses to be encountered both in private and public work. No sooner is the probationer at the end of her training, nay, long before that goal is reached, it becomes of the highest importance to her superiors, her inferiors, and her patients that she should have been taught to think, but often by this time it is too late. She has become accustomed to work by rule of thumb, and she will insist on all who are under her doing the same.

One of the main blessings which the Sister-Tutor has it in her power to confer is the introduction of the art of thinking into the probationer's curriculum. Starting with the physiological facts necessary for her to master, the student of nursing, as distinguished from the nursing aide, finds herself discouraged from mere memorising of text-books and called upon to understand how the various divisions of her subject depend one on the other. All the diagrams and models of the class-room, all the careful demonstrations, all the work done with the microscope, have no other end in the nurse's training than to induce the habit of reflection, comparison, and comprehension. Many women cherish exceeding scorn of the chemical and laboratory work which has been introduced into certain nurse training-schools. They declare that it hampers a nurse in her work to have a smattering of medical education, and tends to make her opinionated. They forget that the surest road to arrogance is to learn by rote and never get a glimpse of the infinite variety of knowledge which lies hidden behind the daily ritual of the hospital ward. Rightly used, there is no better instrument for developing humility as well as powers of thought than intelligent study of some branch of science such as that to which the nurse is introduced in a well-equipped class-room.

But it is in the practical part of the training also that the probationer needs to learn how to think. When a hurried ward sister has to drive women completely unacquainted with their duties through the day's work, always as it were with the point of Time's sword at her throat, she finds little chance of explaining why she issues her various orders and warnings. But when the Sister-Tutor enters to give a lesson to the probationers it is the reasons for the performance of their duties on which she lays stress, and the result is that the probationer learns how to think. More than that, she learns how to make others think, for in passing on the lesson to her junior she is proud to explain why these things are done in this particular way and in no other. In this lies the whole art of teaching.

As experience lessens the rush of work in the hospitals (and we believe the work can be lessened more than any one yet guesses) it may be possible to set probationers to learn to think for themselves by way of observation. Ought they not to be encouraged to watch their patients and form conclusions? Could they not thus learn from the beginning of their training that they exist for the patient's well-being. Under the guidance of a tutor eager to stimulate their intelligence some perception of the patients' trials, some zeal for the healing art, some watchful interest in the whole purpose of hospital endeavour could surely be awakened. The fresh and artless interest of the V.A.D. in those to whom she ministers enters the ward with every new probationer, only to be snuffed out by hurry and professional indifference. In teaching the probationer how to think, it must never be forgotten that women think best when their hearts are stimulated to help their minds.

